# Pharmacology and Regulation of Appetite and Food Intake

**DOI:** 10.1002/prp2.70101

**Published:** 2025-07-28

**Authors:** Fernando Capela e Silva, Elsa Lamy, Ana Margarida Advinha

**Affiliations:** ^1^ Department of Medical and Health Sciences, School of Health and Human Development, MED‐Mediterranean Institute for Agriculture Environment and Development & CHANGE‐Global Change and Sustainability Institute, University of Évora Évora Portugal; ^2^ MED‐Mediterranean Institute for Agriculture Environment and Development & CHANGE‐Global Change and Sustainability Institute, University of Évora Évora Portugal; ^3^ Department of Medical and Health Sciences, School of Health and Human Development, CHRC‐Comprehensive Health Research Centre, LA REAL‐Associated Laboratory in Translation and Innovation Towards Global Health University of Évora Évora Portugal

Food intake is deeply linked to human health and planet sustainability. Lots of non‐communicable diseases are deeply linked to eating behavior. Obesity is recognized by the World Health Organization as a chronic disease that affects several systems and organs and is associated with the development of many other conditions, including high blood pressure, stroke, heart disease, type 2 diabetes, and various types of cancer. Its causes are multiple and generally result from a combination of factors intrinsic to the individual and extrinsic factors, namely, but not limited to, greater availability of high‐calorie and palatable foods and reduced physical activity, which together dynamically influence the increase in energy intake and the decrease in energy expenditure. It is well known that the stigma and discrimination suffered by obese people verified in multiple areas (e.g., work, family, education) can further increase the likelihood of developing mental problems, such as depression, anorexia, bulimia, and others. Obesity is therefore a complex problem whose treatment requires a multidisciplinary approach, representing a serious public health problem that must be prevented and treated. In this sense, it is necessary to know and to understand the physiological systems and control mechanisms that regulate appetite.

Appetite regulation is an extremely complex process, involving functions of the central and autonomic nervous system and the interaction of these with gastrointestinal tract signals, including peptides and hormones. In many cases, the adoption of healthy lifestyles is not enough to control these situations, and it is necessary to resort to the help of psychological or pharmacological treatment (appetite stimulating drugs or appetite suppressants). The food/nutrient–drug interactions also contribute to these phenomena. However, drug safety problems related to excessive weight gain or loss are also a source of concern, and studies of their mechanism and solutions are needed. How eating habits (e.g., food composition, eating regimens) can contribute to an increased risk of adverse effects and vice versa, and how medications can contribute to the emergence of eating disorders are elements requiring careful clinical analysis and risk minimization mechanisms. These can enable contributions to increase user safety and medication/food effectiveness.

This special/virtual issue *Pharmacology and Regulation of Appetite and Food Intake* aimed to bring together high‐quality research and approaches on the physiological systems and control mechanisms that regulate appetite and how to influence and manage the behavioral pathologies associated with it. In the end, nine manuscripts covering important topics of this theme were published: seven reviews, one commentary, and one original research.

In their review, Clarke et al. [1] including research in animal models and in humans, explore therapeutic/pharmacological strategies that have the potential to control food intake. The authors present and discuss the physiological mechanisms that underlie the gut–brain axis and its interaction with the reward network in the regulation of appetite and satiety. In addition to the regulation of food intake and appetite extending beyond the domains of pure physiological need, this work also addresses hedonic mechanisms, including sensory perception (vision, smell and taste), habitual behaviors, and psychological factors as important factors in food intake. Obesity is associated with a complex interaction of several factors, including genetic predisposition and ethnic origins, socioeconomic status, pharmaceuticals, xenobiotics, and sedentary lifestyles with excessive calorie intake and reduced physical activity. It is also worth noting that there is growing evidence that changes in the function and composition of gut microbiota have a crucial role in modulating the host's energy metabolism and consequently in promoting obesity and associated metabolic disorders. In their review article, Rubinić et al. [2] suggest that the gut peptides ghrelin, peptide YY (PYY), and cholecystokinin (CCK) are crucial elements in appetite regulation. The importance of these gastrointestinal peptides in energy balance was evidenced by the changes in their levels after bariatric surgery that seem to be of extreme importance for weight regulation. Thus, the authors suggest these molecules as promising targets for pharmacological interventions in obesity treatment using both peptide‐based and small molecule‐based pharmaceuticals.

The endocannabinoid system (ECS), consisting of cannabinoid receptors together with endocannabinoids, is a complex cellular signaling system responsible for maintaining homeostasis by modulating several regulatory reactions in response to internal and environmental changes. Although the influence of the ECS on the regulation of appetite and energy expenditure has been extensively studied, its real impacts are not yet fully understood. The Cannabinoid type 1 (CB1) receptor is prominent in the central nervous system and the binding of cannabinoids to CB1 has an impact on several physiological processes relevant to the regulation of body weight. In this way, CB1 receptor activation is generally considered a powerful orexigenic signal and inhibition of the ECS is beneficial for the treatment of obesity and related metabolic diseases. Kurtov et al. [3] present several pathophysiological processes in obesity involving the ECS, highlighting different pharmacological options to modulate endocannabinoid activity in the treatment of obesity, although this therapeutic possibility requires further investigation. Additionally, Lord and Noble [4] review the literature to date on exogenous and endogenous cannabinoid action in the hypothalamus with a specific focus on feeding behaviors. The authors highlight the hypothalamic areas on which researchers have focused their attention, including the lateral, arcuate, paraventricular, and ventromedial hypothalamic nuclei, and interactions with the hormone leptin. This review serves as a comprehensive analysis of what is known about cannabinoid signaling in the hypothalamus, highlights gaps in the literature, and suggests future directions on this theme.

Changes in the levels of hormones that regulate food intake, such as leptin, adiponectin, ghrelin and cholecystokinin, can contribute to the development of metabolic diseases at any age due to malnutrition, overweight, lifestyle changes and exposure to extreme environments. Mondal et al. [5] using the in silico approach, identified the key genes that play a crucial role in the leptin signaling pathway. Furthermore, eight miRNAs from the TargetScan 8.0 database were screened out that commonly target these genes. The authors suggest that the role of these miRNAs should be explored, as they may play a vital role in regulating appetite, energy metabolism, metabolic diseases, and combating extreme environments and may be useful in the development of new therapeutics for metabolic diseases, including type 2 diabetes and obesity.

Sato et al. [6] reviewed the mechanisms and pharmacotherapy of cancer cachexia‐associated anorexia. The authors begin by characterizing cachexia, a multifactorial metabolic syndrome, its effects, and the pathologies that may underlie it, including cancer. Considering that some of the current therapeutic strategies to combat it, including the combination of nutritional and exercise interventions, as well as pharmacotherapy that directly affects its pathogenesis, such as anti‐inflammatory agents, metabolism‐enhancing agents, and appetite stimulants, have not proven to be sufficient, the authors refer to new therapeutic agents capable of improving cancer cachexia‐associated anorexia, as well as their mechanisms of action.

Sarcopenia is characterized by a decline in muscle strength, generalized loss of skeletal muscle mass, and impaired physical performance, conditions associated with low quality of life, increased risk of falls, hospitalization, and mortality in the elderly population. Although its development is sustained by aging, there are other factors that can lead to sarcopenia, namely chronic diseases, physical inactivity, inadequate dietary energy intake, and reduced protein intake, leading to an imbalance between the synthesis and degradation of muscle protein. In addition, digestion, absorption, and the ability to metabolize proteins are also altered in aging. On the other hand, evidence emerges that dysbiosis of the intestinal microbiota, due to the effects of various supplements (among other potential factors), directly affects muscle traits, some related to sarcopenia, which suggests the need for a better and more in‐depth understanding of the intestine–muscle axis. Goes‐Santos et al. [7] conduct a narrative review and discuss the evidence for the impact of nutritional supplementation strategies, using animal and vegetable protein, leucine, and creatine, in improving outcomes related to sarcopenia in older adults. The authors also highlight that the results of other supplements, namely branched‐chain amino acids, isolated amino acids, and omega‐3, in sarcopenia are not yet properly proven.

The healthcare model has evolved over the years, considering the patient as the focus of care, seeking to encompass all dimensions in establishing and adapting therapeutic plans. This approach includes a broad and comprehensive review of the medications in those plans, to evaluate, among other considerations, potential drug interactions that may increase possible adverse effects or result in therapeutic failure. In the form of a Commentary, Advinha et al. [8] warn of the need to also evaluate food–drug interactions, integrating this assessment into pharmacovigilance systems. The authors highlight that drug interactions include not only interactions between different drugs but also interactions between drugs and foods, their components, dietary supplements, and herbal products, and that there is an urgent need to develop research that helps health professionals on the implications of potential interactions between foods and drugs. This investigation should include studies on the safety and effectiveness of foods/dietary supplements and analysis of the metabolism of the main active compounds of coexisting drugs. On the other hand, the evaluation of interactions must be developed under conditions that reflect the recommended daily doses and the prescribed frequency, whether of medications or foods.

Early life adversity (ELA) is associated with earlier initiation and maintenance of tobacco smoking and with a greater risk of subsequent relapse. There is growing evidence that appetite hormones, including peptide YY (PYY), which modulates craving and satiety responses, play a role in stress and addiction processes. Through a study that used a quasi‐experimental design, Miller et al. [9] evaluated the association between ELA and circulating PYY stress responses in smokers and nonsmokers to examine the effects of nicotine addiction. The results of this study indicate that experiencing ELA was associated with lower PYY, and no systematic effects of nicotine dependence or relapse were observed. These findings suggest that adults with higher ELA may experience lower PYY. The authors suggest further research to better elucidate the role of PYY in stress and addiction processes. Knowing the causal association between smoking and several diseases and that nicotine can reduce food intake and body weight, the results of this study can also contribute to an integrated knowledge of the molecular mechanisms underlying the correlation between stress and addiction processes, nicotine, food intake/appetite regulators, and body weight/obesity.

## Author Contributions

F.C.S., E.L., and A.M.A. all contributed to the writing and editing of the manuscript.

## Conflicts of Interest

The authors declare no conflicts of interest.

## Data Availability

The authors have nothing to report.

## References

[1] G. S. Clarke , A. J. Page , and S. Eldeghaidy , “The Gut‐Brain Axis in Appetite, Satiety, Food Intake, and Eating Behavior: Insights From Animal Models and Human Studies,” Pharmacology Research & Perspectives 12, no. 5 (2024): e70027, 10.1002/prp2.70027. 39417406 PMC11483575

[2] I. Rubinić , M. Kurtov , and R. Likić , “Novel Pharmaceuticals in Appetite Regulation: Exploring Emerging Gut Peptides and Their Pharmacological Prospects,” Pharmacology Research & Perspectives 12, no. 4 (2024): e1243, 10.1002/prp2.1243. 39016695 PMC11253306

[3] M. Kurtov , I. Rubinić , and R. Likić , “The Endocannabinoid System in Appetite Regulation and Treatment of Obesity,” Pharmacological Research Perspectives 12, no. 5 (2024): e70009, 10.1002/prp2.70009. PMC11409765 39292202

[4] M. N. Lord and E. E. Noble , “Hypothalamic Cannabinoid Signaling: Consequences for Eating Behavior,” Pharmacology Research & Perspectives 12, no. 5 (2024): e1251, 10.1002/prp2.1251. 39155548 PMC11331011

[5] S. Mondal , R. Rathor , S. N. Singh , and G. Suryakumar , “miRNA and Leptin Signaling in Metabolic Diseases and at Extreme Environments,” Pharmacology Research & Perspectives 12, no. 4 (2024): e1248, 10.1002/prp2.1248. 39017237 PMC11253706

[6] R. Sato , G. W. P. da Fonseca , W. das Neves , and S. von Haehling , “Mechanisms and Pharmacotherapy of Cancer Cachexia‐Associated Anorexia,” Pharmacological Research Perspectives 13, no. 1 (2025): e70031, 10.1002/prp2.70031. PMC11707257 39776294

[7] B. R. Goes‐Santos , B. P. Carson , G. W. P. da Fonseca , and S. von Haehling , “Nutritional Strategies for Improving Sarcopenia Outcomes in Older Adults: A Narrative Review,” Pharmacology Research & Perspectives 12, no. 5 (2024): e70019, 10.1002/prp2.70019. 39400516 PMC11472304

[8] A. M. Advinha , J. P. Fernandes , and M. Perdigão , “Food‐Drug Interactions Risk Management: An Emergent Piece of Pharmacovigilance Systems,” Pharmacology Research & Perspectives 12, no. 4 (2024): e1245, 10.1002/prp2.1245. 38992909 PMC11239764

[9] A. A. Miller , M. Nakajima , B. N. DeAngelis , D. K. Hatsukami , and M. al'Absi , “Nicotine Addiction and the Influence of Life Adversity and Acute Stress on PYY: Prediction of Early Smoking Relapse,” Pharmacological Research Perspectives 12, no. 5 (2024): e70016, 10.1002/prp2.70016. PMC11420658 39315578

